# Synthesis and Biological Profile of Omaveloxolone: The Cornerstone for Friedreich Ataxia Treatment

**DOI:** 10.3390/ijms26199747

**Published:** 2025-10-07

**Authors:** Massimiliano Cordaro, Giulia Neri, Shoeb Anwar Mohammed Khawja Ansari, Rocco Buccheri, Angela Scala, Anna Piperno

**Affiliations:** 1 Department of Chemical, Biological, Pharmaceutical, and Environmental Sciences, University of Messina, 31 V.le F. Stagno D’Alcontres, 98166 Messina, Italy; giulia.neri@unime.it (G.N.); shoebanwar.ansari@unime.it (S.A.M.K.A.); ascala@unime.it (A.S.); 2 Department of Drug Sciences, University of Catania, V.le A. Doria, 95125 Catania, Italy; rocco.buccheri@unict.it

**Keywords:** Omaveloxolone, Friedreich’s ataxia, Oleanolic Acid, Ursolic Acid, Bardoxolone, triterpenoid, drug delivery systems

## Abstract

This review provides a comprehensive overview of the therapeutic potential of omaveloxone (OMA) for the treatment of Friedreich’s ataxia (FA), along with an analysis of the historical development and current status of the synthetic strategies for OMA production. OMA activates the nuclear factor-2-(erythroid-2)-related (Nrf2) pathway in vitro and in vivo, in both animal models and humans. The Nrf2 pathway plays a crucial role in the cellular response to oxidative stress. Furthermore, OMA has been shown to mitigate mitochondrial dysfunction, restore redox homeostasis and downregulate nuclear factor-κB (NF-κB), a key mediator of inflammatory responses. Through these mechanisms, OMA contributes to tissue protection and inflammation reduction in patients with FA. The review also highlights future perspective, focusing on the challenges associated with OMA reprofiling through innovative drug delivery approaches and its potential repurposing for diseases beyond FA.

## 1. Introduction

Anomalous inflammation and oxidative stress play a crucial role in many diseases, such as rheumatoid arthritis, chronic lung and kidney diseases, as well as cardiovascular [1] and neurodegenerative diseases [2]. Some studies also correlate them to cancer etiology [3,4]. Consequently, there is an ever-increasing interest in developing novel drugs, such as triterpenoids, that antagonize inflammation and oxidative stress [5,6].

In this review, we focus on synthetic oleanane triterpenoid derivatives which are highly potent anti-inflammatory agents [7]. Recent clinical studies demonstrated promising results for Omaveloxolone (OMA) and its precursor, Bardoxolone (CDDO), triterpenoid electrophilic activators of the Nuclear factor erythroid 2–related factor (Nrf2). Under basal conditions, Nrf2 remains sequestered by its Keap1 inhibitor (Kelch-like ECH-associated protein 1) whereas in oxidative stress conditions or in the presence of exogen activators, such as OMA or CDDO, after dissociation from Keap1, Nrf2 translocates to the nucleus enhancing the expression of genes essential for antioxidant defense, detoxification, and mitochondrial function. By activating Nrf2 and stimulating antioxidant response element (ARE)-driven genes, these synthetic oleanane triterpenoids strengthen cellular defenses against oxidative stress and inflammation, thereby improving cellular resilience [8].

**Figure 1:**
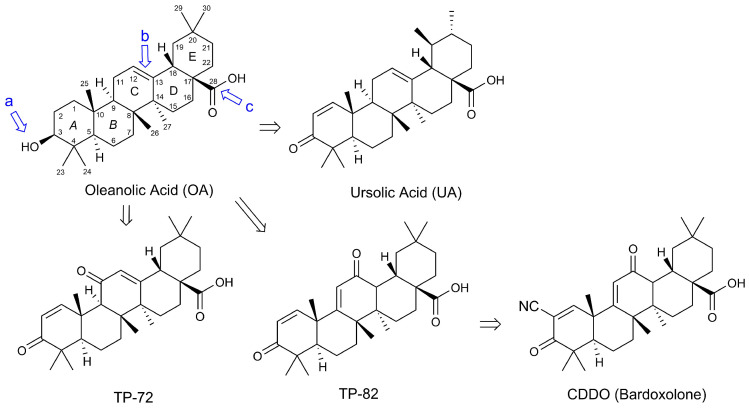
Main “active” portions of Oleanolic Acid (OA): arrow a hydroxyl at C3; arrow b C12-C13 double bond; arrowc carboxylic acid at C28, and key derivatives.

Due to its availability, affordability and intrinsic bioactivity, the starting molecule for synthetic oleanane triterpenoid derivatives is Oleanolic Acid (OA), a pentacyclic triterpene carboxylic acid widely distributed in plants. Through the chemical modification of three reactive sites of OA—the hydroxyl group at C3, the double bond at C12-C13, and the carboxylic acid group at C28 (Figure 1)—numerous new oleanane triterpenoid derivatives have been synthesized. Among these, the oxidation of the hydroxyl group at C3 resulted in A-ring enone derivatives, which paved the way for the creation of a variety of new molecules, such as Ursolic Acid (UA).

Subsequently, the oxidation of the C12-C13 double bond led to the synthesis of C-enone ring derivatives (TP-72 and TP-82) without biological activity. However, the insertion of a deactivating group (cyano) onto the A ring (2-cyano-3,12-dioxooleana-1,9(11)-dien-28-oic acid) led to a compound with significant activity known as Bardoxolone or CDDO (Figure 1) [9].

**Figure 2:**
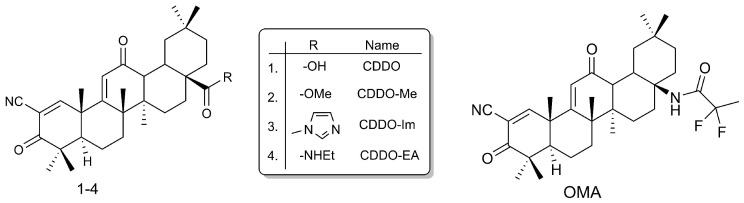
Chemical structures of CDDO derivatives (1−4) and OMA.

CDDO exhibits greater biological activity than UA and several derivatives were synthetized: CDDO-methyl ester (also known as Bardoxolone methyl and RTA 401), CDDO-imidazolide (CDDO-Im), CDDO-ethyl amide (CDDO-EA) (Figure 2) [10,11,12]. Further variations in the functionalization procedures led to the synthesis of a derivative with an amide group, where the nitrogen atom is directly connected to the triterpene ring. The amide contains a three-carbon chain, with two fluorine atoms in the β-position. The chemical name of this molecule is N-((4aS,6aR,6bS,8aR,12aS,14aR,14bS)-11-cyano-2,2,6a,6b,9,9,12a-heptamethyl-10,14-dioxo-1,2,3,4,4a,5,6,6a,6b,7,8,8a,9,10,12a,14, 14a,14b-octadecahydropicen-4a-yl)-2,2-difluoropropanamide, also known as Omaveloxone (OMA) or RTA 408 (Figure 2).

Considering that oxidative stress and inflammation are related to the progression of various diseases, the Kelch-like ECH-associated protein 1-nuclear factor erythroid 2-related factor 2 (Keap1-Nrf2) is widely studied as a key regulator of antioxidant and anti-inflammatory responses [11,12,13,14]. Recent experimental evidence points out that OMA is a potent activator of this Keap1-Nrf2 pathway, more effective than its CDDO precursor [15].

This review summarizes the available knowledge on the therapeutic potential of OMA for the treatment of Friedreich’s ataxia (FA), including the history and the current status of its development for treating neurodegenerative diseases. OMA is able to activate the nuclear factor erythroid 2-related factor 2 (Nrf2) pathway in vitro and in vivo, in both animal models and humans [16]. The Nrf2 pathway plays a crucial role in the cellular response to oxidative stress. There is substantial evidence of suppressed Nrf2 levels and activity in the cells of patients with FA. OMA binds to Kelch-like ECH-Associated Protein 1 (KEAP1), a protein that regulates Nrf2 activity. This binding enables the nuclear translocation of Nrf2 and the transcription of its target genes. In fibroblasts isolated from patients with FA, OMA restores Nrf2 protein levels and enhance Nrf2 activity. Furthermore, OMA mitigates mitochondrial dysfunction and restores redox homeostasis in these cells, as well as in neurons from mouse models of FA [17]. In addition, OMA not only enhances the effects of Nrf2 but also downregulates nuclear factor-κB (NF-κB) activity, a key mediator of inflammatory response. Thus, OMA contributes to tissue protection and inflammation reduction in patients with FA [18]. Overall, OMA is a promising therapeutic option for the treatment of neurological diseases, including FA, due to its direct activation of the Nrf2 pathway [19,20]. In 2023, the FDA approved OMA as the first treatment for FA in individuals aged 16 and older. OMA’s safety and effectiveness in treating FA were evaluated in a 48-week, double-blind, randomized, placebo-controlled clinical trial (NCT02255435) [21,22]. This review provides an overview of the latest OMA research findings, focusing on the chemical and biological aspects and its future pharmacological and therapeutic prospects.

## 2. Chemistry of Omaveloxolone and Its Precursors

Triterpenoids are widely distributed in nature, and within each class, there is significant structural diversity and a broad range of biological activities [23]. Research often focuses on derivatives that are commercially available and easily obtainable from natural sources. The derivatization of these precursors through synthetic procedures has become a well-established area of study, particularly in recent decades. Thanks to this research, new biologically active molecules have been developed, with synthetic oleanane triterpenoids being representative examples [24]. For instance, UA was prepared first, followed by Bardoxolone (CDDO), which is the precursor of OMA [25].

This category of triterpenoid molecules exhibits structural similarities to steroids with some important differences in their chemical reactivity. Among these differences, the most evident is the presence of hydroxyl at C3 and carboxyl at C28 adjacent to quaternary carbons. This neopentyl-type functionalization is known for being particularly inert to nucleophilic attack (S_N_2) due to steric hindrance. As a result, both C3 and C28 are highly unreactive to substitution reactions, primarily because of the difficulty that nucleophiles have in approaching these sites due to the presence of two geminal methyl groups at C4 and the fusion of D and E rings. These structural features limit the reactivity at C-3 and C-28 with secondary cellular nucleophiles and make chemical functionalization challenging.

It has been demonstrated that the functionality of the Michael reaction acceptor is critical for biological activity. For this reason, natural OA, which lacks electrophilic functionality, is inactive towards certain targets (NAD(P)H-quinone acceptor oxidoreductase) [26]. In contrast, CDDO derivatives (Figure 2), which feature a highly electrophilic cyanoenone group on ring A, exhibit favorable chemical interactions with the nucleophilic functional groups of amino acid residues in target proteins. Specifically, reversible thio-Michael adducts are formed with cysteine residues, which compete with the formation of aza-Michael adducts involving cellular amines, thereby reducing their toxicity.

Hetero-Michael reactions (e.g., thio-, aza-, and oxa-Michael reactions) have gained significant importance in organic synthesis, especially for biological applications, due to their unique reversibility properties [27,28].

In this framework, we believe it is useful to summarize the most recent methods for the preparation of OA, CDDO and OMA to provide guidelines for the design of new analogous derivatives.

### 2.1. Oleanolic Acid Extraction, Characterization and Biological Activity

Pharmacologically active compounds are typically present in plants at low concentration, making their recovery and purification from raw materials dependent on suitable extraction processes [29]. Extraction methods for OA include Soxhlet extraction, ultrasound-assisted extraction, and microwave-assisted extraction, among others [30]. The characterization and identification of OA have been accomplished using analytical techniques such as nuclear magnetic resonance (NMR), thin-layer chromatography (TLC), and High-Performance Liquid Chromatography (HPLC) [31]. The main methods used for OA extraction are described in Table 1, along with the variability in extraction yields, botanical sources and biological properties. Notably, the biological effects attributed to OA span multiple therapeutic areas, ranging from anti-inflammatory and anticancer properties to antimicrobial qualities [30,32,33,34,35,36,37,38,39,40,41,42,43]. Numerous studies reported that triterpenoids in plants are concentrated in the intracuticular wax layer [44]. As a result, the OA content is significantly higher in the “skin” or “peel” of the fruit (non-botanical terms that encompass the cuticle and various cell types) compared to the pulp. Table 2 highlights the distribution of OA within different fruit tissues, revealing a consistent trend of higher concentrations in external layers such as skin and peel. The variability in OA levels across species and tissue types also points to the influence of genetic and environmental factors on triterpenoid biosynthesis. This pattern supports the hypothesis that OA accumulation is linked to the presence of cuticular waxes and suggests that whole-fruit consumption, particularly with peel, may enhance dietary intake of this compound [45].

### 2.2. Bardoxolone Synthesis

**Scheme 1:**
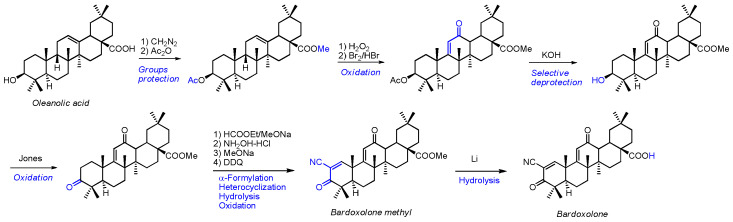
Synthetic strategy for Bardoxolone (CDDO) as reported by Honda et al. [25].

The introduction of a 1-en-3-one moiety and an electron-withdrawing group (i.e., nitrile functionality) into the triterpenoid ring A significantly enhances the biological activity of the resulting new derivatives compared to the natural OA precursor (Figure 1). In 2000, the synthesis of CDDO was achieved through an 11-step synthetic procedure, with an overall yield of 29% [25]. The strategy involved the selective oxidization of rings A and C through a series of protection/deprotection steps (Scheme 1).

**Scheme 2:**
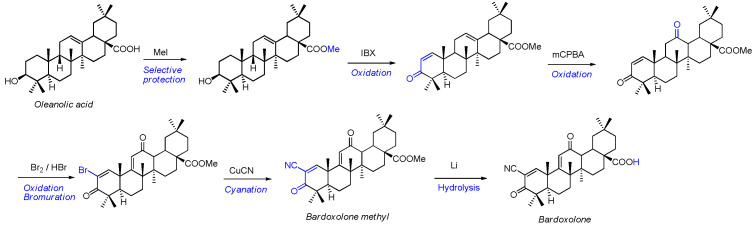
Synthetic strategy for CDDO optimized by Fu et al. [46].

In 2013, Fu et al. reported a modified and more efficient approach reducing the synthetic process to six steps [46] (Scheme 2).

Recent studies [47] on CDDO derivatives adopted the improved synthetic strategy that led to the development of a wide range of CDDO derivatives, primarily as ester derivatives (Figure 2) with improved pharmacological properties. In the last 10 years, approximately 350 articles, 120 patents, and 75 reviews have been published on this topic, highlighting the biological activity and therapeutic potential of these compounds [47,48].

**Figure 3:**
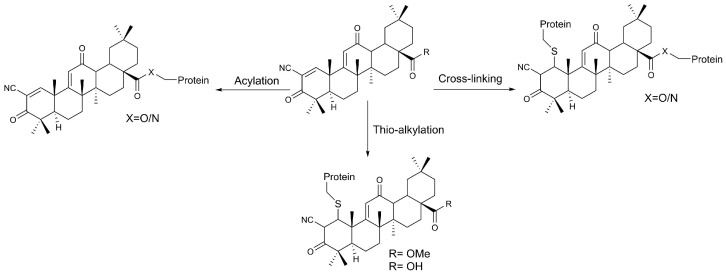
Pathways for the Formation of Triterpenoid–Protein Adducts.

The ester moiety plays a crucial role in the interaction with proteins. Literature data demonstrate differential reactivity of CDDO derivatives with nucleophilic amino acid residues on target proteins. For instance, the triterpenoid CDDO-Im forms covalent adducts and cross-links even at low concentrations (10–50 nM), whereas CDDO-Me does not form such adducts even at higher concentrations (500 μM) (Figure 3) [49].

### 2.3. Chemistry of Omaveloxolone

Omaveloxolone (OMA) is a CDDO derivative obtained via transesterification, introducing an amide functionality at the C-17 position of the triterpenoid scaffold (Figure 2).

To date, no experimental details have been disclosed in scientific literature, as the synthetic procedure was patented by Reata Pharmaceuticals, Inc. (Irving, TX, USA) in 2013 [50] and more recently by Sicor Societa Italiana Corticosteroidi S.r.l. in 2024 [51]. Here, we summarize the synthetic approach reported in the patents to provide an overview of the employed methodologies and to provide the scientific community an organic chemist’s perspective on the state of the art of OMA synthesis.

**Scheme 3:**
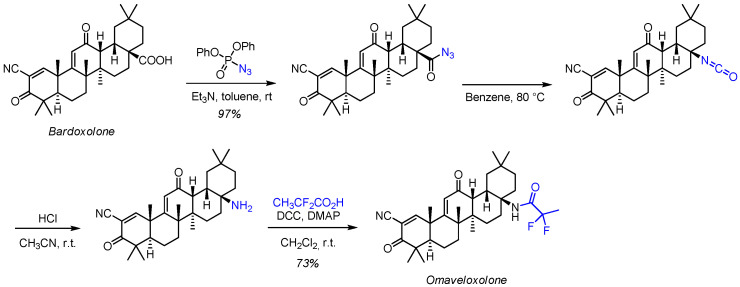
Synthetic route to OMA as described in [52].

OMA (Figure 2) preserves the characteristic oleanane core, including a fused five-ring system, which is crucial for maintaining molecular rigidity and three-dimensional conformation required for biological activity [22]. Although the structure-activity relationship (SAR) of OMA has not yet been fully elucidated, the presence of electrophilic functionalities (cyano group, enone moiety, and carbonyl groups) suggests a strong potential for interaction with Keap1 and subsequent activation of Nrf2 [48]. The first synthesis of OMA (Scheme 3) [52] involves a four-step sequence using CDDO as starting material, with a good overall yield.

The synthetic approach involves the preparation of an acyl azide intermediate, followed by a Hofmann rearrangement to generate an isocyanate, which is subsequently hydrolyzed to an amine. This was then coupled with a carboxylic acid under standard amide-forming conditions. However, the use of azides poses safety concerns due to their explosive potential particularly on a large scale. Additionally, the intermediate is obtained as a non-pure enantiomeric form, requiring chromatographic purification to obtain the final product.

**Scheme 4:**
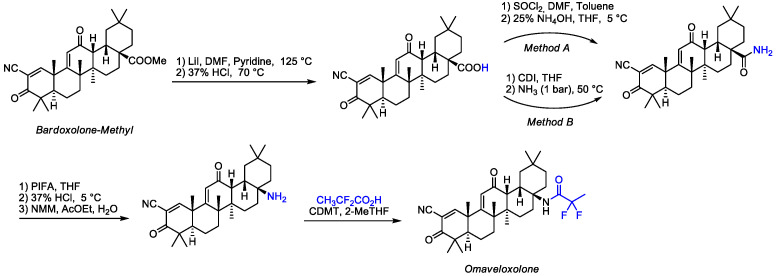
Improved synthetic strategy for OMA [51].

To address these limitations, Sicor Società Italiana Corticosteroidi (Milan, Italy) recently patented a more efficient and safer synthetic method for OMA [51], which avoids the use of azides and the need for chromatographic purification. and promotes the formation of solid, easily handled intermediates. The synthetic route for OMA (Scheme 4) involves Hofmann’s rearrangement to convert amides into amines, yielding a pure final product. This four-step synthetic procedure can be streamlined to three steps by using (bis(trifluoroacetoxy)iodo)benzene (PIFA) for the amide-to-amine transformation. followed by coupling with 2,2-difluoropropanoic acid under mild conditions (Scheme 4).

This approach offers significant advantages in terms of safety and scalability.

The transformation of the amide derivative into the amine was evaluated with various reagents, including bromine or chlorine in the presence of an alkali metal hydroxide, preferably sodium hydroxide; sodium hypochlorite or hypobromite; lead tetraacetate in the presence of triethylamine; N-bromosuccinimide in the presence of 1,8-diazabicyclo [5.4.0]undec-7-ene (DBU), (PIFA), or (diacetoxyiodo)benzene (PIDA). Preferred solvents included diethyl ether, tetrahydrofuran, 2-methyltetrahydrofuran, diisopropyl ether, acetone, ethyl acetate, cyclohexanone. The amine derivative was isolated by solvent addition and treatment with an organic base (pyridine, pyrrolidine, N-methylpyridine, N-methylpiperidine, morpholine, or N-methylmorpholine (NMM)). The temperature was chosen based on the type of transformation, as shown in Table 3.

The spectroscopic characterization of OMA is reported in references [50,51]. The proton NMR spectrum shows three downfield signals at 8.7 ppm (s), 7.8 ppm (s), and 6.3 ppm (s), corresponding to the NH-amide protons, C-H(1), and C-H(11), respectively. In the upfield region, the protons C-H(13) and C-H(18) appear at 3.4 ppm and 3.1 ppm, respectively, and all other protons resonate between 2.0 and 0.9 ppm.

## 3. Drug Delivery Systems of Omaveloxolone and Its Precursors

OMA and its CDDO and OA precursors, due to their triterpenoid nature, are characterized by low water solubility (Table 4) that significantly affects key parameters, such as pharmacokinetics, pharmacodynamics, drug distribution, protein binding, and absorption [47]. Currently, OMA is administered as an oral once-daily capsule with an immediate release. The main drug delivery challenges include low aqueous solubility, food effects (administration on an empty stomach is recommended), and pediatric swallowing. At the best of our knowledge, no literature papers describe nanoformulations or drug delivery systems (DDS) loaded with OMA, although several innovative formulation strategies have been developed for its precursors to enhance solubility, bioavailability, and therapeutic efficacy. Currently, the development of efficient DDS remains a significant challenge for several marketed bioactive compounds, including antitumoral, antimicrobial, and neurological drugs [53,54,55,56].

A comprehensive review by Wasim et al. [57] discussed various approaches aimed at enhancing OA’s biopharmaceutical features. Table 5 provided a selection of recent representative OA and CDDO nanoformulations that include advanced biological evaluations, highlighting their respective merits and limitations.

## 4. Computational and Statistical Techniques

Computational models and statistical techniques are increasingly pivotal in Friedreich’s Ataxia (FA) research, particularly in assessing the impact of emerging therapeutics such as OMA. These methods contribute to all stages of disease evaluation, from understanding disease mechanisms to optimizing clinical trial design and drug development. Their relevance is especially pronounced in the study of rare diseases, where limited patient populations constrain the availability of extensive clinical data. Computational approaches help bridge these gaps by integrating heterogenous data sources, enabling more robust and reliable analyses.

### 4.1. Model-Informed Drug Development (MIDD)

The development and approval of OMA for FA exemplify the potential of Model-Informed Drug Development (MIDD), particularly in overcoming or addressing challenges associated with rare diseases. This approach relies on integrating natural history data into clinical trials, thereby addressing the limitations of conducting large-scale, controlled studies.

A key statistical method used in the evaluation of OMA was logistic regression, which facilitated the estimation of propensity scores to match patients in the MOXIe extension study with external control data from the FA Clinical Outcome Measures Study (FACOMS). This matching process, based on variables such as sex, age, and baseline modified FA Rating Scale (mFARS) scores, enabled researchers to more accurately isolate the drug’s effect from the natural progression of the disease. The mFARS scoring system is a well-established metric for measuring the severity and progression of neurological symptoms in FA. The study found that patients treated with OMA demonstrated significant improvements in mFARS scores, supporting the drug’s efficacy.

Integrating external control data and natural history datasets provided a stronger evidentiary foundation for regulatory approval, demonstrating the potential of computational techniques to generate reliable clinical insights even in contexts with limited patient populations. Additionally, the use of open-label extension studies combined with external controls enabled a more comprehensive evaluation of OMA safety profile. This case highlights how MIDD techniques can expedite drug development for rare diseases by utilizing advanced statistical analyses and data modeling to inform evidence-based therapeutic decisions [64].

### 4.2. Physiologically Based Biopharmaceutics Modeling (PBBM)

Physiologically Based Biopharmaceutics Modeling (PBBM) was employed to investigate the effects of food intake on the pharmacokinetics of OMA. PBBM integrates drug substance properties, formulation characteristics, and physiological parameters to predict drug absorption and distribution within the body.

Researchers first constructed a physiologically based pharmacokinetic (PBPK) absorption model using both intravenous and oral data from non-human primates. This model was then extrapolated to human subjects, incorporating critical parameters such as drug solubility, permeability, and the influence of systemic drug transporters, such as P-glycoprotein (P-gp), on drug efflux. Additionally, in vitro metabolic clearance data were used to refine the model’s accuracy.

Model validation was achieved by comparing predictions against clinical study results. Sensitivity analyses were performed to identify key factors influencing OMA absorption and to determine the main sources of variability. The model successfully predicted observed pharmacokinetic behaviors, revealing unexpected trends. While a linear correlation between peak plasma concentration (Cmax) and total drug exposure (AUC) is typically expected, the study found that administering OMA with a high-fat meal resulted in a 350% increase in Cmax, accompanied by only a 15% increase in AUC.

The model explained this phenomenon by showing that drug absorption is limited by solubility in the fasted state. However, increased bile salt solubilization in the fed state enhances dissolution and absorption in the upper gastrointestinal tract. This, in turn, increases first-pass metabolism in the gut, accounting for the disproportionate rise in Cmax relative to AUC. These findings underscore how PBBM contributes to elucidate complex pharmacokinetic behaviors and can inform optimized drug administration strategies to improve therapeutic efficacy [65].

### 4.3. Wearable Devices and Machine Learning

Traditional clinical scales for monitoring FA progression often suffer from subjectivity, low sensitivity to subtle disease changes, and prolonged evaluation periods. To address these limitations, researchers have explored the use of wearable devices combined with machine learning models to track disease progression more objectively and efficiently.

Two independent research groups demonstrated the potential of wearable motion sensors to predict clinical scores associated with FA. These studies involved collecting kinematic data, including the time spent sitting, standing, and walking, as well as movement speed and complexity. This real-time data was then used to train machine learning algorithms, demonstrating a higher predictive accuracy for disease progression than traditional clinical scales.

By capturing nuanced movement patterns that might escape standard clinical assessments, these AI-driven models could significantly reduce the duration and size of clinical trials for FA and other neurodegenerative diseases. The ability to detect small yet meaningful changes in motor function enables a more responsive and precise evaluation of treatment efficacy, ultimately expediting the drug development process. This approach represents a promising shift towards digital biomarkers and AI-assisted diagnostics, paving the way for more personalized and data-driven patient care [66,67].

### 4.4. Future Directions

The integration of computational and statistical techniques in FA research is still in the process of evolving. Future advancements may include:

- Multi-Omics Integration: Combining genomic, transcriptomic, and proteomic data with computational models to uncover novel biomarkers and therapeutic targets.
- Digital Twins: Developing patient-specific digital twins to simulate disease progression and predict individual treatment responses.
- Real-World Evidence (RWE): Expanding the use of real-world data from wearable devices and electronic health records to enhance the generalizability of research findings.

Furthermore, we wish to highlight the current gap in SAR knowledge, identifying it as a promising avenue for future computational and pharmaceutical chemistry research. Addressing this limitation will not only emphasize the importance of SAR exploration but also lay the groundwork for more targeted and effective drug discovery efforts.

By embracing these future directions, the field of FA research can continue to leverage cutting-edge computational and statistical methodologies to accelerate treatment development, improve patient outcomes, and ultimately move toward a more personalized and practical approach to disease management.

## 5. Conclusions

This review highlights the current state of the art in OMA research and the recent advances in its synthetic strategies. The approval of OMA by international regulatory agencies, in 2023, marked a major milestone in addressing the significant unmet medical needs of patients with FA demonstrating proven efficacy in slowing disease progression, sustained long-term benefits, and a favorable safety profile. OMA’s unique mechanism provides broad cellular protection that extends beyond direct antioxidation, establishing it as a landmark therapy in FA. Its repurposing has been proposed to leverage mitochondrial, antioxidant, and anti-inflammatory effects for other conditions, including mitochondrial myopathies, neurodegenerative disorders, and rare diseases such as Ataxia-Telangiectasia. However, research outside of FA remains largely at the preclinical or early exploratory stage (computational, in vitro, or animal models). Advancing its use in humans will require rigorous clinical trials to establish safety, optimal dosing, and efficacy.

Future research should prioritize refining synthetic methodologies to expand the portfolio of OMA derivatives and enhance the scalability of chemical processes. Moreover, the field of drug delivery for OMA remains largely unexplored, with only a few examples of its precursors, CDDO and OA, reported in the literature so far. There is significant potential to exploit the synergy between nanotechnology and artificial intelligence, both for repurposing OMA and for driving the development of efficient, innovative OMA-based drug delivery systems.

## Figures and Tables

**Table 1:** Natural sources of OA, extraction method and biological properties.

Plant Sources	Extraction (Yield)	Biological Activity	Ref.
*Olea europaea*	Maceration (90%)	Antitumor, antimicrobial, anti-diabetic	[32,33]
*Ligustrum lucidum*	Microwave assisted extraction (5.8 mg/gm)	Anti-hepatitis, anti-inflammatory, antioxidative, antitumor, antiprotozoal, antimutagenic	[30,34]
*Syzygium aromaticum/Clove*	Soxhlet extraction (92%)	Antinociceptive, antioxidant, anti-inflammatory, antihypertensive	[35,36]
*Lantana camara*	Maceration in ethanol 1.74% dry weight of root	Anti-inflammatory, antioxidative, antiprotozoal	[37]
*Achyranthes aspera*	Microwave and ultrasonic assisted extraction, (1.1% to 1.9%)	Antimicrobial, anti-inflammatory	[38]
*Satureja montana*	Percolation with diethyl ether (1.9%)	Antitumor, antibacterial	[39]
*Aspilia Africana*	Cold maceration (56.78%)	Anti-inflammatory, Antioxidant	[40]
*Monotheca buxifolia*	Maceration (0.5% to 0.9%)	Antipyretic	[41,42]
*Vitis vinifera*	maceration (0.5% to 1%)	Antibacterial, antitumor	[43]

**Table 2:** Concentrations of OA in fruits (dm, dry matter; dw, dry weight; fw, fresh weight).

Fruits	Analyzed Part	OA Concentration
Olives	Skin	3094–4356 µg/g fw
	Pulp	27–29 µg/g fw
Bilberries	Whole fruit	1679.2–2029.6 µg/g dw
Jujube	Pulp	360 ± 10.7 µg/g dw
Pears	Skin	164.3–3066.6 µg/g dw
	Pulp	34.0–156.0 µg/g dw
Grapes	Peel	176.2 µg/g dw
Pomegranate	Peel	26.96 ± 0.93 µg/g dw
	Seed	1.12 ± 0.09 µg/g dw
Apples	Pomace	16 µg/g.dm
	Skin	28 µg/g.dm
Lemon	Peel	0.62 ± 0.01 µg/g dw

**Table 3:** Reaction conditions for OMA synthesis using different reagents and protocols.

Patent	Reagents	Conditions
[50]	Step (a) Diphenyl phosphoryl azide (DPPA) Step (b) Benzene Step (c) Sodium sulfate, sodium bicarbonate Step (d) CH_3_CF_2_CO_2_H, DCC, DMAP	Solvents: Toluene, Triethylamine, Ethyl acetate, Chloroform Step a: 0 °C for 5 min, then r.t. overnight Step b: 80 °C with stirring for 2 h Step c: Acetonitrile, cooled to 0 °C Step d: Methylene chloride, Hexanes, Room temperature (25 °C)
[51]	Step (a) Br_2_/NaOH, Cl_2_/NaOH, NaOCl, NaOBr, Pb(OAc)_4_/Et_3_N, NBS/DBU, PIFA, PIDA Step (b) DCC or CDMT, DMAP or NMM	Solvents: Diethyl ether, THF, Me-THF, DMF, DMSO, etc. Step a: 40–180 °C depending on reagent PIFA/PIDA: 5–30 °C Step b: −10 to 15 °C

**Table 4:** Physicochemical properties of OA, CDDO and OMA.

Parameters	OA	CDDO	OMA
Molecular Formula	C_30_H_48_O_3_	C_32_H_43_NO_4_	C_33_H_44_F_2_N_2_O_3_
Molecular weight	456.7 g/mol	505.7 g/mol	554.71 g/mol
Solubility	Insoluble in H_2_O,	Insoluble in H_2_O	Insoluble in H_2_O
	21 mg/mL in DMSO	20 mg/mL in DMSO	≥55.5 mg/mL in DMSO
	7 mg/mL in Ethanol	10 mg/mL Ethanol	≥25.05 mg/mL in Ethanol
pKa	4.74	-	7.4
Appearance	Solid	Solid Powder	Solid
Color	White	White to yellow	White to off-white

**Table 5:** Comparative table of selected nanoformulations of OMA precursors with their advanced biological evaluations.

**OA**		
**Ref**	**Description**	**Comment**
Wei et al. 2023 [58]	Galactosylated chitosan-modified liposomes (GC@Lipo) were loaded with OA to address the challenge of non-specific drug distribution in hepatocellular carcinoma (HCC) treatment.	GC@Lipo binds to the asialoglycoprotein receptor (ASGPR) on HCC cell surfaces, improving the antitumor efficacy of OA. In mouse Hepa1-6 cells, OA loaded GC@Lipo inhibited migration and proliferation, upregulating E-cadherin and downregulating N-cadherin, vimentin, and Anexelekto compared to free OA.
Chen at al 2025 [59]	Targeted hybrid liposomes were loaded with OA and fused with neutrophil membrane coating (N-OAL) to enhance the precision treatment of renal ischemia–reperfusion injury	N-OAL enhances the accumulation and retention at inflammatory sites associated with AKI through biomimetic chemotaxis mediated by neutrophil membranes specifically targeting damaged renal tubular epithelial cells. N-OAL exerts significant antioxidant, anti-inflammatory anti-apoptotic properties. The remarkable protective effect of N-OAL on oxidative-damaged renal tissue caused by AKI induction was confirmed in vivo.
Zang 2025 [60]	OA nanoparticles (138.1 nm) were obtained by emulsion solvent evaporation method for the treatment of ulcerative colitis. Their anti-inflammatory effects and therapeutic efficacy were evaluated in vitro (RAW264.7 cells) and in vivo (DSS-induced UC mouse model).	OA NPs reduced oxidative stress and inflammation by downregulating TNF-α, IL-6, and IL-1β pro-inflammatory cytokines and promoting macrophage polarization from M1 to M2. Oral administration of OA NPs significantly alleviated colitis symptoms, reduced inflammation, and mitigated tissue damage, improved colon morphology, with minimal systemic toxicity.
**CDDO**		
**Ref**	**Description**	**Comment**
He 2024 [61]	Nanostructured lipid carriers were loaded with CDDO and conjugated with anti-PECAM-1 antibody (PECAM@CDDO NLCs) to specifically bind pulmonary vascular endothelial cells that highly express PECAM-1 receptors for the treatment of acute lung injury (ALI).	PECAM@BM NLCs accumulated in the lungs and significantly alleviated the inflammation of ALI. PECAM@BM NLCs inhibited the assembly of NLRP3 inflammasome and pro-caspase-1 complex, thereby suppressing the induction of pyroptosis and resulting in the inhibition of N-terminal GSDMD expression without systemic toxicity.
Huang 2024 [62]	CDDO was incorporated into peptide amphiphile micelles containing RGD peptide to bind the basolateral surface of renal tubules via integrin receptors for treating polycystic kidney disease. Four drug combinations in RGD micelles were evaluated (CDDO, Octreotide, Salsalate, Pravastatin).	The highest synergistic effects were observed between CDDO and Salsalate in both 2D and 3D autosomal dominant polycystic kidney disease (ADPKD) in vitro models. Both combinations CDDO/Octreotide and CDDO/Salsalate in RGD micelles conferred greater therapeutic benefits in ADPKD mice.
Ye 2024 [63]	Targeted CDDO self-assembled nanoparticles conjugated with p28 peptide and loaded with lexiscan (LEX) (p28-LBM NPs) were designed for glioblastoma (GBM) treatment	p28-LBM NPs successfully penetrated brain tumors after intravenous administration, significantly inhibited GBM tumor growth and extended the survival of mice with tumors.

## Data Availability

No new data were created or analyzed in this study.
